# Spatial transcriptomic approaches for characterising the bone marrow landscape: pitfalls and potential

**DOI:** 10.1038/s41375-024-02480-8

**Published:** 2024-11-28

**Authors:** Rosalin A. Cooper, Emily Thomas, Anna M. Sozanska, Carlo Pescia, Daniel J. Royston

**Affiliations:** 1 Nuffield Division of Clinical Laboratory Sciences, Radcliffe Department of Medicine, University of Oxford, Oxford, UK; 2 Oxford University Hospitals NHS Foundation Trust, Oxford, UK; 3 Institute of Biomedical Engineering, University of Oxford, Oxford, UK; 4 University of Milan, Milan, Italy

**Keywords:** Preclinical research, Cancer imaging

Recent years have seen the rapid emergence of spatial transcriptomic (ST) technologies that provide spatially resolved expression-based descriptions of tissue architecture. These approaches can offer a high degree of transcriptional coverage, providing novel opportunities for high-resolution characterisation of tissue microenvironments. Whilst there is now an extensive literature providing benchmarking for quality control (QC) of ST data [1–5], these workflows have exclusively focussed on downstream analysis of expression data without reference to other critical QC steps, namely assessment of cellular morphology, tissue integrity and architectural preservation. We recognise the need for wider discussion of these aspects to inform reliable and accurate ST analysis. Here, we outline existing approaches for spatially-resolved profiling of the bone marrow (BM) and the potential for ST analysis in this context. Drawing on our experience of ST analysis of human BM [6] we also present a workflow for the preparation of bone marrow trephine (BMT) material and integration with H&E morphology to support reliable downstream analysis, outlining potential pitfalls. Our insights have implications beyond the assessment of BMT material and provide a framework of good practice for generating and analysing ST data.

## Existing approaches for spatially profiling the bone marrow

The bone marrow (BM) in adulthood is the main site of haematopoiesis and as such provides a wealth of information regarding haematopoietic health. A comprehensive understanding of haematopoiesis is dependent on spatially-resolved descriptions of the BM, with haematopoietic stem and progenitor cell (HSPC) niches defined by their spatial location in relation to bone and vascular structures [7]. Conventional spatially-resolved profiling of the BM has relied on insights drawn from H&E morphology, or limited panels of either immunohistochemical (IHC) or immunofluorescent (IF) markers. Sarachakov et al. [8] used multiplex IF to describe remodelling of the ageing marrow, with HSPCs consistently spatially located with respect to bone, but showing subtle morphological variation with ageing. Frequently, insights into cell-cell interaction in the BM are drawn from single cell RNA-seq (scRNA-seq) datasets, with in-situ localisation of a candidate marker explored with IHC or IF. For example, recent work by Bandyopadhyay et al. [9] used a CODEX panel [10, 11] informed by a stromal cell-enriched scRNA-seq dataset of human BM cells to characterise human BM architecture, describing a peri-adipocytic HSPC niche, and localisation of early myeloid progenitor populations within the endosteal niche. The CODEX technology utilises antibody-bound DNA oligonucleotides that undergo fluorescent labelling, providing single-cell identification for multiplexed tissue-based imaging [10, 11]. Whilst IF-based approaches capture a limited number of probe targets, IF-multiplexing can provide targeted phenotyping of multiple cells and subpopulations of interest. In addition, these approaches are typically more cost-effective than ST platforms and have the additional benefit of providing protein-based expression profiling. However, probe-based ST approaches offer opportunities to more comprehensively characterise BM microenvironmental features, providing truly comprehensive whole-section in-situ phenotyping of all cell populations, subpopulations, and interrogation of cellular differentiation trajectories and biological pathway activation. ST approaches are also not subject to limitations posed by lack of staining specificity, or stearic hindrance affecting marker selection [12].

## The potential and pitfalls of spatial transcriptomic approaches

ST approaches for mapping the BM microenvironment have the potential to enhance our understanding of human haematopoiesis and characterise mechanisms underpinning haematological neoplasia. Advances in ‘omic’ techniques including scRNA-seq have transformed our understanding of haematopoiesis and the cellular evolutionary perturbation underpinning blood cancer [13]. Spatial cellular organisation provides complementary insights into tissue biology and function [14], with histological assessment of a BMT using haematoxylin and eosin (H&E)-stained sections a key part of the diagnostic pathway. ST approaches can potentially bridge the gulf between molecular insights and conventional H&E-based morphology. These approaches provide more comprehensive quantitative descriptions than those afforded by lower-plex methods, and can capture the full repertoire of cellular diversity, maturation and differential activation within the BM. ST analysis can also provide quantification of the BM microenvironment without the BM sampling biases inherent within aspirate samples, which are typically enriched for haematopoietic cells and depleted for mesenchymal stromal cell populations [9].

There are now multiple commercial platforms providing in-situ transcriptomic profiling, each with advantages and limitations. Commercial ST platforms are either sequencing-based (*Nanostring GeoMx [Seattle, WA]; 10x Visium [Pleasanton, CA]*), or imaging-based, utilising either probe-based or in-situ sequencing technology (*Vizgen MERSCOPE [Cambridge, MA]; NanoString CosMx [Seattle, Washington]; 10x Xenium* [15, 16]). Sequencing-based platforms provide up to whole-transcriptome coverage, although output is typically limited to selected regions of interest (ROI) (*GeoMx*) or a spot-based spatial matrix (*Visium*). Neither of these platforms provide single-cell resolution or whole-section coverage, but do offer near whole-transcriptome expression profiling [17]. These approaches are therefore particularly well suited for hypothesis generation and differential expression analysis, with quantitative assessment of differential abundance possible with deconvolution (via packages such as *Cell2location* [18], *Stereoscope* [19] *and SPOTlight* [20]*)* using a suitable reference dataset. In addition to these commercial platforms, techniques such as DBiT-seq [21] and Slide-seq [22] provide high resolution sequencing-based ST profiling, albeit just short of cellular resolution. Imaging-based platforms (*Xenium; MERSCOPE*) provide whole sample analysis at single cell resolution. These platforms are of limited plex, albeit many orders of magnitude greater than those provided by most protein-based approaches [17]. At present, these platforms offer pre-designed probe sets and variable options for customisation. The *10x Xenium* platform offers several pre-designed panels suitable for specific tissues of interest (e.g. Human Lung, Mouse Brain), with options for full customisation of up to 480 probes. The *Vizgen MERSCOPE* platform also offers pre-designed panels of 500 markers and options for panel customisation of up to 1000 probes. These platforms are most suited for capturing a population of interest or expression of a candidate gene marker. However, ongoing technical advancements now mean that increasingly larger, expanded panels are becoming available, for example the *10x Xenium* Prime 5k, and *Nanostring CosMx* 6k panels. A more comprehensive technical comparison of these and other spatial omic platforms can be found elsewhere [23–25].

Despite the widespread use of these commercial ST platforms to characterise microenvironmental features in several tissue types, there are few examples of successful in-situ expression-based profiling of BM material [25]. Groups who have successfully utilised such analyses have employed in-situ bulk RNA-seq approaches [26–28]. Baccin et al. [26] used laser capture microdissection and sequencing of the murine marrow with subsequent deconvolution informed by a scRNA-seq dataset to describe the spatial localisation of CXCL12-abundant reticular (CAR) cells. Xiao et al. [27] used *Visium* to define spatial patterns of biological pathway activation across the murine marrow, and identified a skeletal stem and progenitor cell niche. Notwithstanding its relative novelty, the paucity of groups successfully generating ST BM data may partly reflect the technical challenges associated with handling BMT material, including difficulties in obtaining consistent, high-quality sections, and impaired nucleic acid preservation following decalcification. Whilst the potential of ST is increasingly clear, there are evidently barriers to the widespread application of these technologies to BM material.

## A framework of good practice for tissue preparation and integration with H&E morphology for spatial transcriptomic analysis

**Fig. 1:**
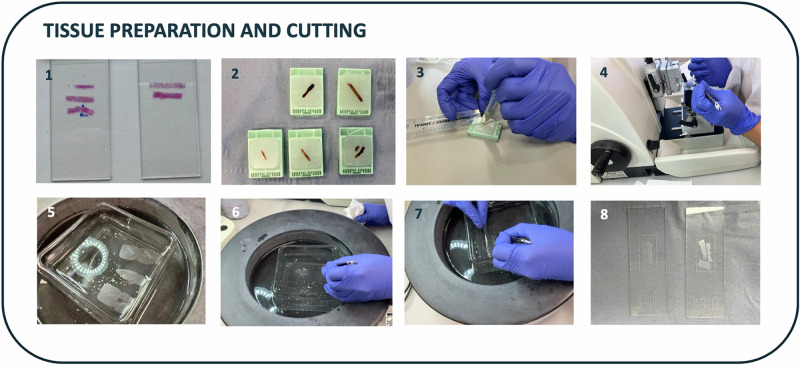
Exemplar framework for good practice in tissue preparation and sectioning. A stepwise approach for tissue preparation and sectioning for spatial transcriptomic (ST) analysis (FFPE sections of BMT tissue for *10x Xenium* analysis). Prior to sectioning the environment should be cleaned with RNaseZap to ensure a RNase free environment. 1. Pathology review of a H&E-stained section. 2. Review FFPE tissue blocks to ensure macroscopic BMT tissue integrity. 3. Gently and very lightly score around the BMT region of interest with a scalpel, excluding areas not suitable for ST analysis e.g. thick cortical bone/haemorrhage. 4. Chill and rehydrate the FFPE block in an ice bath and then cut and discard the first few sections until a full-face of the BMT is seen and surface paraffin is removed. Cut a thin (see relevant protocol for thickness) section from the FFPE block with a microtome. 5. Immediately transfer the tissue section to surface of a warm (42°C or as per protocol) water bath using tweezers, careful to touch only the peripheral paraffin rather than tissue. Lie the section flat on the surface of the water and inspect the section for integrity and absence of folding. 6. Without touching the BMT tissue, remove the excess paraffin from the section with tweezers and discard. The light scoring in step three will facilitate removal of excess paraffin whilst preserving the BMT tissue. 7. Manoeuvre the BMT sections (on the surface of the water) onto the appropriate region of the ST slide. Placing the ST slide in the water bath under the tissue sections minimises the need to touch the tissue during this step. 8. Allow the tissue to dry on the slide as per protocol to be taken forward for further processing. Refer to the relevant user manual for further details including regarding water bath preparation, block chilling, drying temperature and timing.

Frameworks outlining good practice for quality control (QC) and analysis of ST datasets are manifold but almost exclusively outline bioinformatic processing including cellular segmentation, cellular annotation and spatial clustering [1–5]. The impact of upstream tissue preparation and quality on downstream ST data analysis is almost entirely unacknowledged. By contrast, the importance of image and section quality in clinical pathology workflows is well-recognised and is a routine step in generating a diagnostic H&E section. More recently, quality assessment has been shown to be a key step to facilitate the application of artificial-intelligence (AI)-based algorithms for pathology in large clinical cohorts [29], with systematic strategies for automating quality assessment of tissue sections developed to improve algorithm validation and advance clinical adoption [30]. However, this process has largely been limited to clinical pathology and biomedical engineering communities. Drawing on our own recent experience of ST analysis of human archival formalin-fixed paraffin-embedded (FFPE) BMTs, we regard such considerations as equally important for ST analytical workflows. Here we highlight two main steps in the ST workflow where expert H&E morphological review is critical; the first upstream of tissue sectioning, and the second following generation of ST data. Whilst providing a detailed technical protocol is outside the scope of this article, we outline a process for optimising tissue sectioning of BMT material for ST analysis that highlights potential pitfalls (Fig. 1).

### Morphological review of H&E and tissue sectioning

The first step of any ST experiment should involve pathological review of a high-quality H&E section (Fig. 1). This will confirm specimen suitability and allow identification of regions of architectural and morphological preservation for targeted macrodissection. Even in experienced hands, BMT sections deemed adequate for clinical pathological assessment may contain artefacts such as crush, haemorrhage and folding and may not be suitable for ST analysis. At this stage, sections can be cut for RNA extraction and assessment of RNA quality to identify the sample DV200 (percentage of RNA fragments >200 base pairs in length) or RIN (RNA integrity) score. However, caution should be exercised as other features such as block storage and delayed fixation affect assay sensitivity [31]. In addition, for imaging-based platforms (*Xenium*), correlation between DV200 and median transcript count per cell may be poor [32]. Following initial morphological review, a thin section of either FFPE or frozen tissue is cut, which is then transferred to a water bath prior to being immediately manoeuvred onto an ST slide (Fig. 1). Limited tissue handling during this step minimises tissue disruption and reduces the risk of tissue detachment downstream. Lightly scoring the FFPE blocks prior to sectioning facilitates macrodissection of selected regions in the water bath without direct contact with the BMT material (Fig. 1). BMTs are bony specimens and require decalcification prior to sectioning, which impairs nucleic acid preservation [33, 34]. Decalcification with ethylenediaminetetraacetic acid (EDTA) minimises degradation of nucleic acid quality and facilitates nucleic-acid probe-based assays such as fluorescence in-situ hybridisation (FISH) compared to decalcification with hydrochloric or formic acids [34, 35]. To maximise nucleic acid preservation, EDTA decalcification of BMT material using a suitable and consistently-applied protocol may be preferable for ST analysis.

### Integration of ST output with H&E morphology

**Fig. 2:**
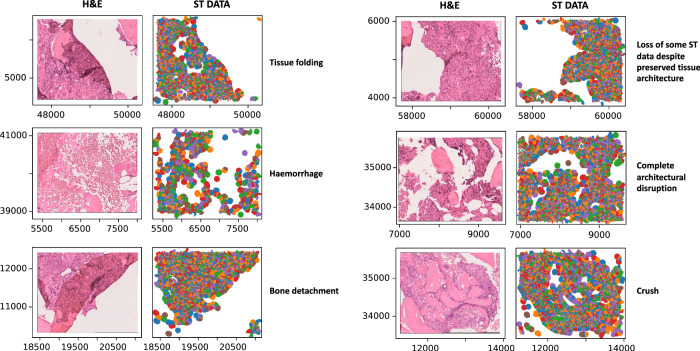
Comparing ST data with H&E-stained tissue morphology. H&E images show areas of crush, haemorrhage, architectural disruption, tissue folding and bone detachment which are not readily identifiable on review of the spatial transcriptomic (ST) data output. The right column shows the ST data output (each coloured circle represents a cell) and left column the corresponding H&E image for the same region. x and y axes indicate spatial coordinates.

**Fig. 3:**
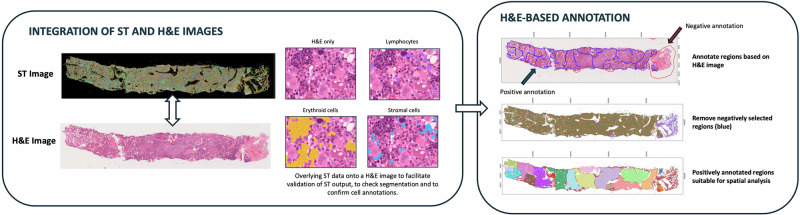
Integration of ST data with H&E images. Left panel: Following generation of spatial transcriptomic (ST) data using the Xenium platform, H&E staining can be performed on the same section. The ST data can be integrated with the H&E image and be visualised overlying the H&E morphology. This step allows for confirmation of architectural preservation and cellular morphology, as well as visual confirmation of appropriate cellular segmentation. The panel shows H&E image of a BMT, with three distinct cell groups highlighted (purple, blue and yellow indicating lymphocytes, stromal and erythroid groups respectively, clockwise from top right). Right panel: H&E-based annotation. (Top and middle BMTs) Regions in which cell annotation is compromised (e.g. due to haemorrhage or crush) should be negatively annotated for removal from downstream analysis. Areas of architectural preservation are positively annotated for subsequent spatial analysis. (Bottom BMT) Each discrete positively annotated region (here representing an intertrabecular space) is indicated by a different colour.

We have recently generated a large ST BMT cohort from human archival FFPE tissue sections using the *10x Xenium* platform [6]. Crucially, we find that the ST image output is not a reliable representation of underlying tissue preservation, and that areas of crush, haemorrhage, bone detachment, tissue folding, and architectural disruption are not reliably identified on inspection of the ST data (Fig. 2). These factors may affect analysis in two ways: firstly, by impairing confidence in cellular annotation, with transcripts erroneously attributed during segmentation; and secondly, in the inappropriate application of spatial analysis to assess microenvironmental features in regions of poor architectural preservation. We also occasionally find areas of tissue preservation on H&E with no corresponding ST output (Fig. 2). Processing and sampling induced tissue artefacts that disrupt the spatial integrity of the sample are a particular challenge in BMT material. ST data should therefore not be analysed agnostic to H&E morphology. With tissue preserving platforms such as *Xenium*, overlying the ST output on an H&E image derived from the same section facilitates validation of architectural preservation whilst also supporting lineage assignment (Fig. 3). This also supports validation of ST cellular segmentation, which in cells with large polylobated nuclei such as megakaryocytes may not be accurate and requires further refinement.

To mitigate the influence of tissue artefact we suggest that prior to downstream analysis a process of initial H&E-guided ‘negative selection’ is performed to identify and remove regions in which cell identification and lineage assignment is compromised (e.g. regions of crush and tissue folding) (Fig. 3). This should be followed by a process of ‘positive selection’ to identify regions of architectural preservation within which spatial analysis is appropriate. As spatially-resolved description of cellular organisation is dependent on the assumption of preserved tissue architecture representative of the in-vivo microenvironment, we suggest that the presentation of results should routinely include release of H&E-stained images alongside quantitative ‘omic’ data and that this should be considered the gold standard for ST-based work. This is particularly important for BMT tissue, where tissue quality may be compromised due to the technical challenges associated with sampling and processing.
